# Depletion of PD-1 or PD-L1 did not affect the mortality of mice infected with *Mycobacterium avium*

**DOI:** 10.1038/s41598-021-97391-4

**Published:** 2021-09-09

**Authors:** Masayuki Nakajima, Masashi Matsuyama, Mio Kawaguchi, Sosuke Matsumura, Takumi Kiwamoto, Yosuke Matsuno, Yuko Morishima, Kazufumi Yoshida, Mingma Thsering Sherpa, Kai Yazaki, Ryota Tanaka, Naoko Okiyama, Masafumi Muratani, Yukio Ishii, Nobuyuki Hizawa

**Affiliations:** 1 Department of Respiratory Medicine, University of Tsukuba, 1-1-1 Tennoudai, Tsukuba, Ibaraki 305-8575 Japan; 2 Department of Dermatology, University of Tsukuba, Ibaraki, Japan; 3 Department of Genome Biology, Faculty of Medicine, University of Tsukuba, Ibaraki, Japan

**Keywords:** Infection, Immunology, Microbiology

## Abstract

The programmed cell death-1 (PD-1) and programmed cell death-ligand 1 (PD-L1) pathway could affect antimicrobial immune responses by suppressing T cell activity. Several recent studies demonstrated that blocking of the PD-1/PD-L1 pathway exacerbated *Mycobacterium tuberculosis* infection. However, the effect of blocking this pathway in pulmonary *Mycobacterium avium–intracellulare* complex (MAC) infection is not fully understood. Wild-type, PD-1-deficient mice, and PD-L1-deficient mice were intranasally infected with *Mycobacterium avium* bacteria. Depletion of PD-1 or PD-L1 did not affect mortality and bacterial burden in MAC-infected mice. However, marked infiltration of CD8-positive T lymphocytes was observed in the lungs of PD-1 and PD-L1-deficient mice compared to wild-type mice. Comprehensive transcriptome analysis showed that levels of gene expressions related to Th1 immunity did not differ according to the genotypes. However, genes related to the activity of CD8-positive T cells and related chemokine activity were upregulated in the infected lungs of PD-1 and PD-L1-deficient mice. Thus, the lack of change in susceptibility to MAC infection in PD-1 and PD-L1-deficient mice might be explained by the absence of obvious changes in the Th1 immune response. Furthermore, activated CD8-positive cells in response to MAC infection in these mice seemed to not be relevant in the control of MAC infection.

## Introduction

The prevalence of pulmonary non-tuberculous mycobacterial (NTM) disease has increased significantly worldwide^1^. *Mycobacterium avium–intracellulare* complex (MAC) accounts for 90% of pulmonary NTM. Although the factors predisposing to pulmonary NTM disease have not been fully elucidated, several lines of evidence have implied that defects of T cell immunity may underlie progression of pulmonary NTM^2^. Lower production capacity of IFN-γ and TNF-α induces MAC disease progression^3^. In addition, TNF-α blocking agents, such as infliximab and adalimumab, are known to exacerbate pulmonary NTM disease^4^.

Immune checkpoint inhibitors (ICIs) have recently shown efficacy against many malignant tumors and are being widely used^5^. Programmed cell death-1 (PD-1) is one of these immune checkpoint molecules, which is predominantly expressed on activated CD4-positive T cells and CD8-positive T cells^6^. Binding of PD-1 with its ligand programmed cell death-ligand 1 (PD-L1) leads to the activation of the PD-1/PD-L1 pathway, and it regulates the immune response by suppressing T cell immunity. In fact, the PD-1/PD-L1 pathway also plays a major role in CD8-positive T cell exhaustion during chronic viral infections and cancer^7^. Although the role of PD-1 in mycobacterial infection has not been fully elucidated, several recent studies have shown that blocking the PD-1/PD-L1 pathway exacerbates *Mycobacterium tuberculosis* infection. For example, PD-1 deficient (*PD-1*^*−/−*^) mice demonstrated increased bacterial burden and inflammation following *M. tuberculosis* infection^8^. Treatment with a PD-1/PD-L1 inhibitor exacerbated *M. tuberculosis* infection in patients with lung cancer^9^. In contrast, bacterial numbers in the spleen of *PD-1*^*−/−*^ mice were significantly reduced compared with wild-type mice 6 and 12 weeks after *Mycobacterium bovis* bacillus Calmette–Guérin (BCG) infection^10^. Fujita et al. reported 3 cases of acute *Mycobacterium avium* complex lung disease during immunotherapy with ICIs^11^, whereas Ishii et al. suggested that nivolumab had a positive effect in the treatment of *Mycobacterium abscessus* lung disease^12^. In the present study, the effect of depletion of the PD-1/PD-L1 pathway in pulmonary MAC infection was investigated using a mouse model.

## Results

### Depletion of PD-1/PD-L1 did not affect mortality and bacterial burden in mice infected with MAC

**Figure 1:**
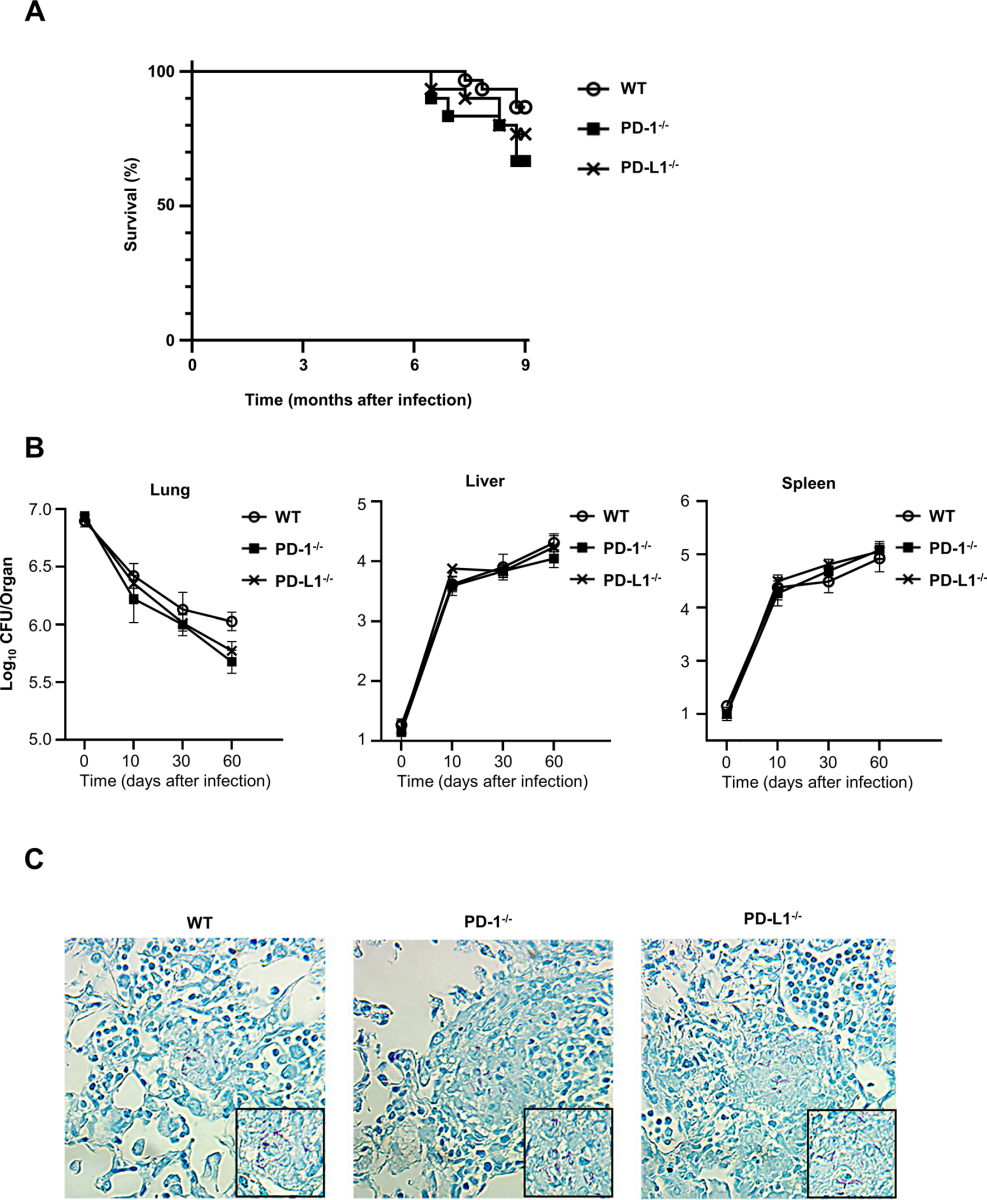
Depletion of PD-1/PD-L1 did not affect susceptibility to *M. avium*. (**A**) Survival of wild-type, *PD-1*^*−/−*^, and *PD-L1*^*−/−*^ mice after intranasal inoculation of 1 × 10^7^ CFU of MAC or saline (n = 30 in each group). (**B**) Mycobacterial outgrowth in the lung, liver, and spleen of wild-type, *PD-1*^*−/−*^, and *PD-L1*^*−/−*^ mice at 0, 10, 30, and 60 days after intranasal inoculation of 1 × 10^7^ CFU of MAC. The results are expressed as CFU per organ. The experiments were performed in duplicate with eight mice in each group. Data are expressed as means ± SEM. Significance was defined as a P value of < 0.05. (**C**) Representative photographs of Ziehl–Neelsen staining of lungs (× 400). Insets show acid-fast bacilli at higher magnifications.

To assess the effect of depletion of the PD-1/PD-L1 pathway in pulmonary MAC infection, the survival of wild-type, PD-1 deficient (*PD-1*^*−/−*^), and PD-L1 deficient (*PD-L1*^*−/−*^) mice after inoculation of MAC was investigated. Unlike the previous reports of tuberculosis^8,9^, the survival rate was not different among these genotypes (Fig. 1A). In addition, mycobacterial growth in lung, liver, and spleen was not different among the genotypes at any time point (Fig. 1B). Acid-fast bacilli were detected in granuloma-like lesions (Fig. 1C). When a lower amount of MAC inoculation (1 × 10^5^ CFU) was tested, mycobacterial growth in lung was again similar in these 3 genotypes 60 days after MAC inoculation (Figure E1A). These results indicated that depletion of the PD-1/PD-L1 pathway did not affect the pivotal immune response against MAC infection, at least in this mouse model.

### Depletion of the PD-1/PD-L1 pathway enhanced MAC-induced pulmonary lymphocytic inflammation

**Figure 2:**
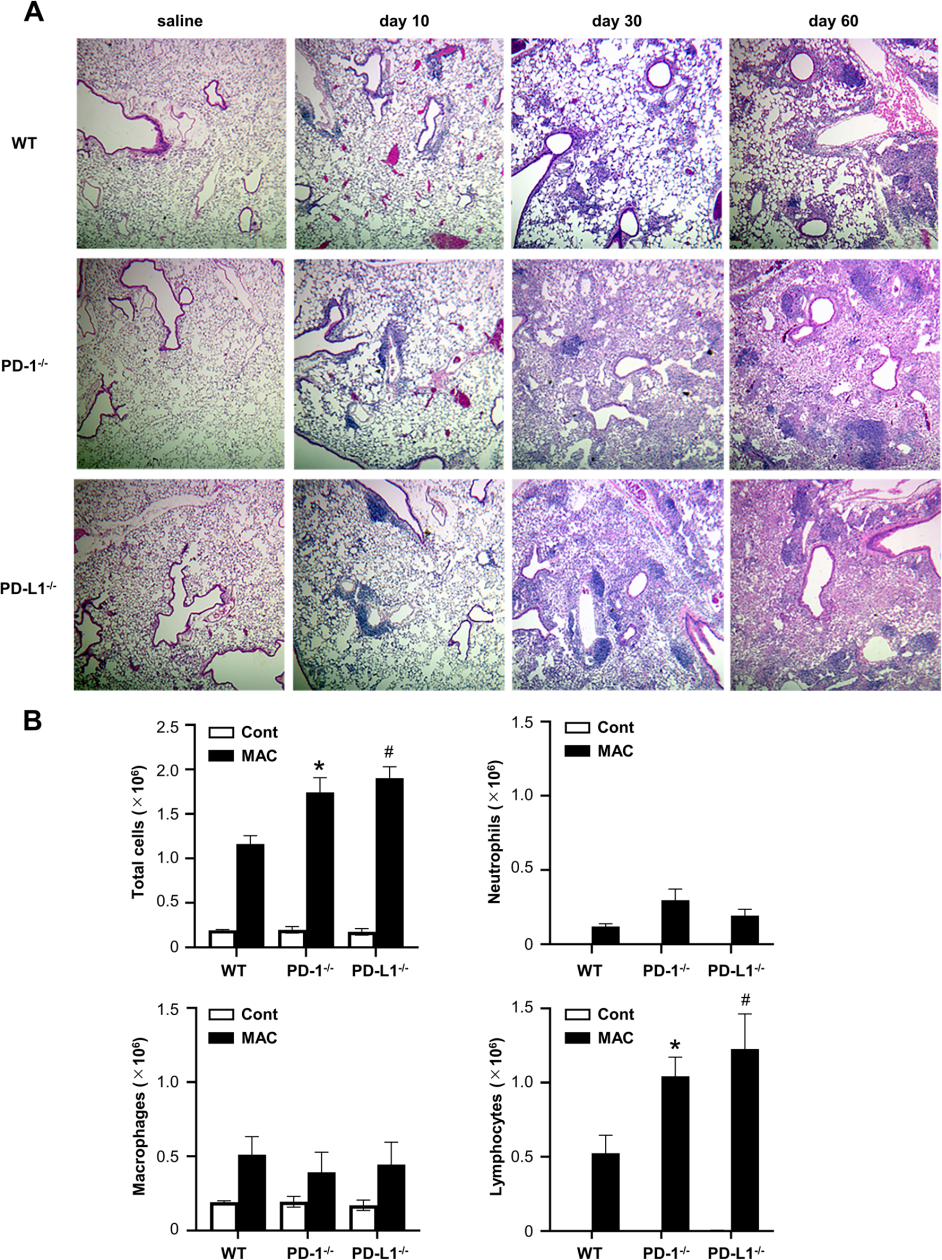
Depletion of PD-1/PD-L1 enhanced MAC-induced pulmonary inflammation. (**A**) Representative photomicrographs of lungs from wild-type, *PD-1*^*−/−*^, and *PD-L1*^*−/−*^ mice at 10, 30 and 60 days after intranasal inoculation of 1 × 10^7^ CFU of MAC or saline. Hematoxylin and eosin stain (× 40). (**B**) Numbers of total cells, neutrophils, macrophages, and lymphocytes in BAL fluids of wild-type, *PD-1*^*−/−*^, and *PD-L1*^*−/−*^ mice 60 days after intranasal inoculation of 1 × 10^7^ CFU of MAC. Control mice were administered saline (open bars). The experiments were performed in duplicate with four mice in each group. Data are expressed as means ± SEM. Significance was defined as a P value of < 0.05. *Significant difference between wild-type mice and PD-1^*−/−*^ mice (P < 0.05). ^#^Significant difference between wild-type mice and *PD-L1*^*−/−*^ mice (P < 0.05).

Inflammatory cell infiltration was observed in peribronchial and perivascular regions after MAC infection, but not in the saline-treated group (Fig. 2A). Inflammatory cell infiltration was marked in the lungs of *PD-1*^*−/−*^ and *PD-L1*^*−/−*^ mice compared with wild-type mice 30 and 60 days after MAC infection. The numbers of BAL-recovered inflammatory cells, including macrophages, neutrophils, and lymphocytes, were increased in all genotypes 60 days after MAC infection (Fig. 2B). The numbers of lymphocytes were significantly higher in *PD-1*^*−/−*^ and *PD-L1*^*−/−*^ mice than in wild-type mice at that time point. When a lower amount of MAC was inoculated (1 × 10^5^ CFU), the numbers of lymphocytes were again significantly higher in *PD-1*^*−/−*^ and *PD-L1*^*−/−*^ mice (Figure E1B).

### Depletion of the PD-1/PD-L1 pathway caused infiltration of CD8-positive T cells

**Figure 3:**
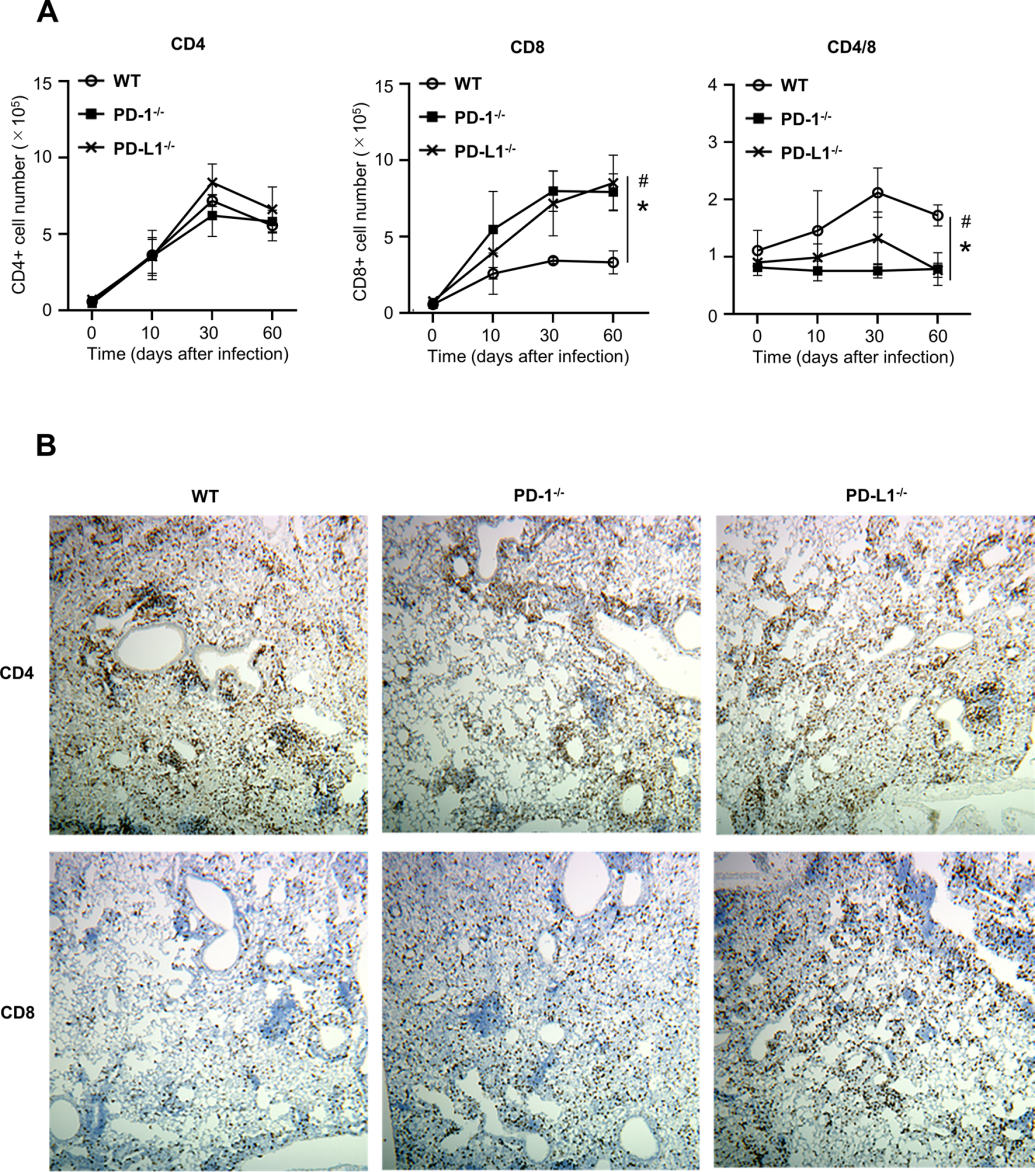
Pulmonary inflammatory cell analysis of wild-type, *PD-1*^*−/−*^, and *PD-L1*^*−/−*^ mice after MAC infection. (**A**) The numbers of CD4 and CD8-positive cells in the lungs of wild-type, *PD-1*^*−/−*^, and *PD-L1*^*−/−*^ mice 0, 10, 30, and 60 days after intranasal inoculation of 1 × 10^7^ CFU of MAC. The ratio of CD4/CD8 was also calculated. (**B**) Representative photographs of immunostaining for CD4 and CD8 in the lungs of wild-type, *PD-1*^*−/−*^, and *PD-L1*^*−/−*^ mice 60 days after MAC infection (× 100). The experiments were performed in duplicate with four mice in each group. Data are expressed as means ± SEM. Significance was defined as a P value of < 0.05. *Significant difference between wild-type mice and *PD-1*^*−/−*^ mice (P < 0.05). ^#^Significant difference between wild-type mice and *PD-L1*^*−/−*^ mice (P < 0.05).

Using flow cytometry analyses, the numbers of CD4-positive T cells and CD8-positive T cells were investigated in the lungs of wild-type, *PD-1*^*−/−*^, and *PD-L1*^*−/−*^ mice on day 60 after MAC inoculation. As shown in Fig. 3A, the numbers of CD4-positive T cells and CD8-positive T cells were increased in response to MAC infection in all genotypes. The numbers of CD8-positive T cells, but not CD4-positive T cells, were significantly higher in the lungs of *PD-1*^*−/−*^ and *PD-L1*^*−/−*^ mice than in those of wild-type mice 60 days after MAC infection.

**Figure 4:**
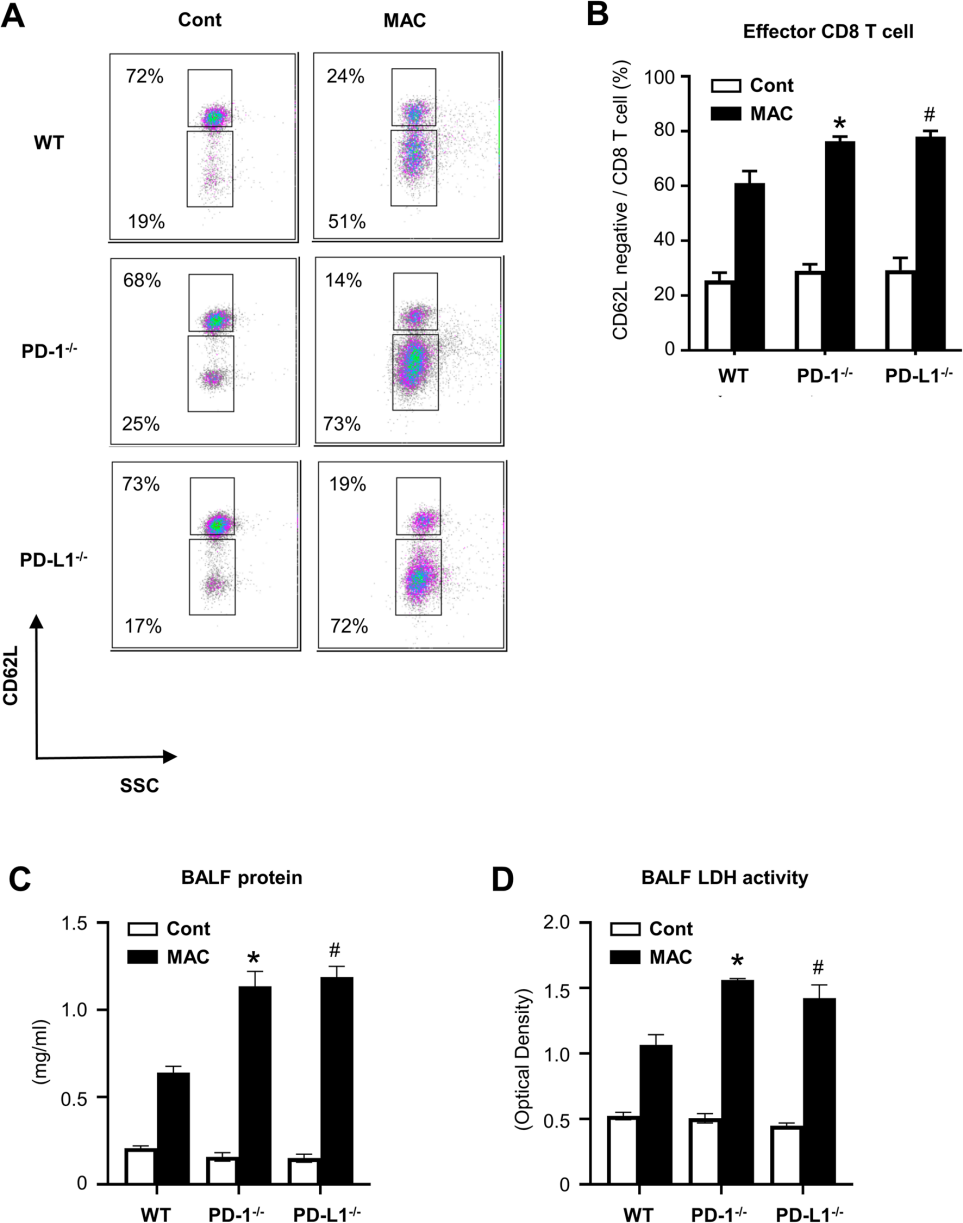
Analysis of CD8-positive T cells in the lungs after MAC infection. (**A**) The proportions of CD62L-negative cells in CD8-positive cells obtained from lungs of wild-type, *PD-1*^*−/−*^, and *PD-L1*^*−/−*^ mice 60 days after intranasal inoculation of 1 × 10^7^ CFU of MAC or saline (Cont). Representative plots are shown. CD8-positive T cells were analyzed for their cell surface expression of CD62L. (**B**) The mean proportions of four samples are shown. (**C**) The concentrations of total protein in BAL fluids of wild-type, *PD-1*^*−/−*^, and *PD-L1*^*−/−*^ mice 60 days after intranasal inoculation of 1 × 10^7^ CFU of MAC. (**D**) LDH cytotoxicity in BAL fluids of wild-type, *PD-1*^*−/−*^, and *PD-L1*^*−/−*^ mice 60 days after intranasal inoculation of 1 × 10^7^ CFU of MAC. The experiments were performed in duplicate with four mice in each group. Data are expressed as means ± SEM. Significance was defined as a P value of < 0.05. *Significant difference between wild-type mice and *PD-1*^*−/−*^ mice (P < 0.05). ^#^Significant difference between wild-type mice and *PD-L1*^*−/−*^ mice (P < 0.05).

CD4-positive T cells were accumulated in the peribronchial region and the center of granulomas in all genotypes 60 days after MAC infection (Fig. 3B). In *PD-1*^*−/−*^ and *PD-L1*^*−/−*^ mice, CD8-positive T cells were predominantly located in the alveolar region 60 days after MAC infection (Fig. 3B). To evaluate the activity of CD8-positive T cells in the infected lungs of *PD-1*^*−/−*^ and *PD-L1*^*−/−*^ mice, CD62L expression was analyzed in these CD8-positive T cells (Fig. 4A,B). The proportion of CD8-positive and CD62L-negative T cells was significantly higher in the lungs of *PD-1*^*−/−*^ and *PD-L1*^*−/−*^ mice than in those of wild-type mice (Fig. 4B), suggesting that CD8-positive T cells in the infected lungs of *PD-1*^*−/−*^ and *PD-L1*^*−/−*^ mice are in the effector phase. In addition, the protein and LDH levels of BAL fluids were significantly higher in the infected lungs of *PD-1*^*−/−*^ and *PD-L1*^*−/−*^ mice than in those of wild-type mice, which may reflect the increased cytotoxicity of CD8-positive T cells (Fig. 4C,D).

**Figure 5:**
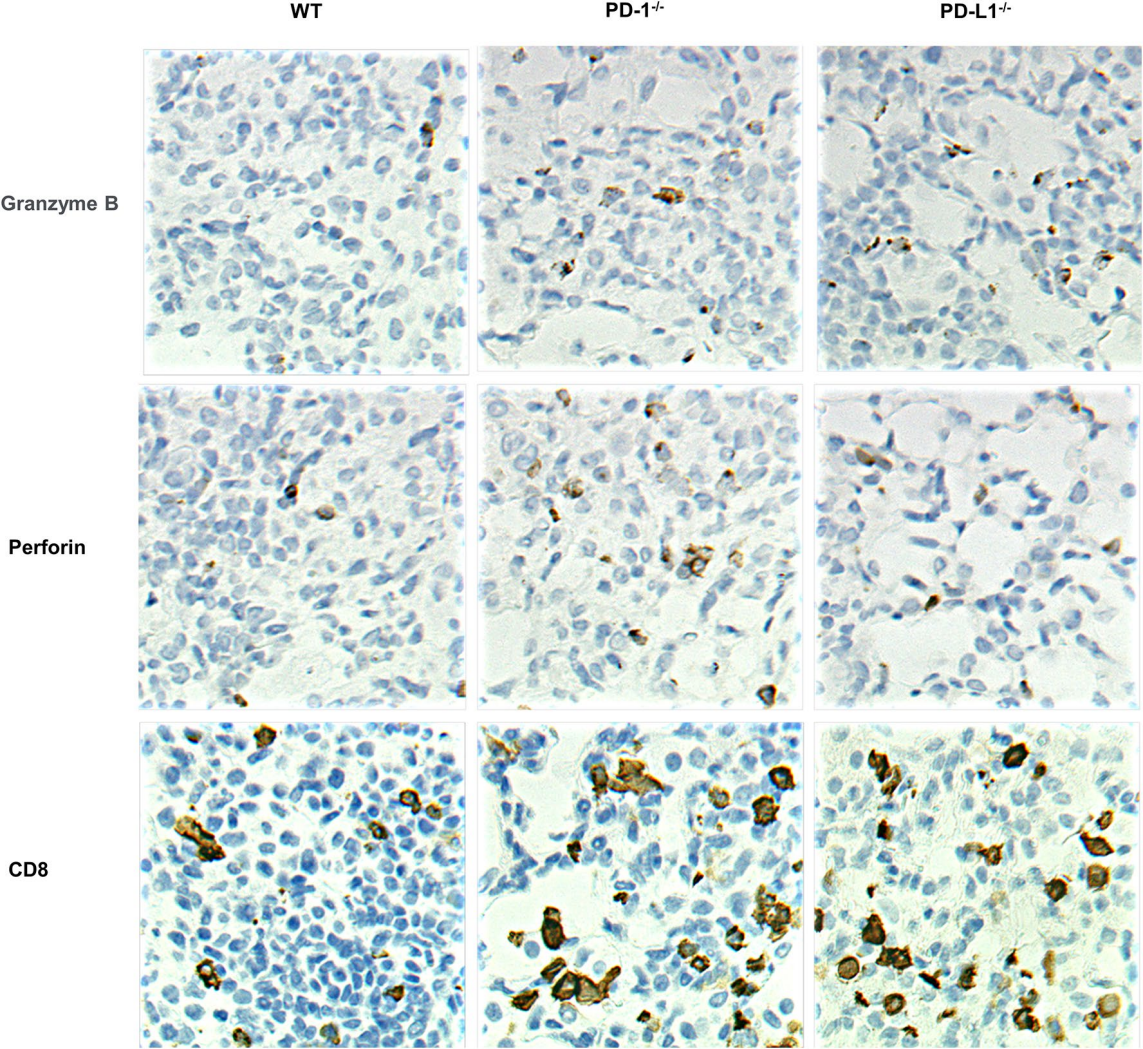
Pathogenic role of CD8-positive T cells in the lungs after MAC infection. Representative photographs of immunostaining for CD8, granzyme B, and perforin in the lungs of wild-type, *PD-1*^*−/−*^, and *PD-L1*^*−/−*^ mice 60 days after MAC infection (× 400).

To evaluate the cytotoxicity of CD8-positive T cells, granzyme B and perforin immunostaining was performed on the lungs 60 days after MAC infection. As shown in Fig. 5, granzyme B and perforin were expressed in some CD8-positive T cells in the infected lungs. Higher numbers of granzyme B-positive and perforin-positive cells were observed in the infected lungs of *PD-1*^*−/−*^ and *PD-L1*^*−/−*^ mice than in those of wild-type mice, indicating that the cytotoxic activity of CD8-positive T cells was enhanced in the infected lungs of *PD-1*^*−/−*^ and *PD-L1*^*−/−*^ mice.

Since IFN-γ plays a central role in protection against mycobacterial pathogens, whether CD4-positive or CD8-positive T cells contribute to IFN-γ production against MAC bacteria from the lungs of wild-type, *PD-1*^*−/−*^, or *PD-L1*^*−/−*^ mice was examined. IFN-γ-producing CD4-positive and CD8-positive T cells increased in the lungs of all mice after MAC infection (Figure E2A,B). However, there was no significant difference among genotypes. These results indicated that IFN-γ production by CD4-positive and CD8-positive T cells after MAC infection was not affected by the PD-1/PD-L1 pathway.

### RNA-seq analyses showed that depletion of the PD-1/PD-L1 pathway did not affect the gene expressions related to activated Th1 immunity, but increased gene expressions related to CD8-positive T cells in response to MAC infection

To investigate the effects of differential expression of mRNA transcripts on infection with MAC bacteria in each genotype, a comparison analysis was performed using RNA-seq data. Sixty days after MAC inoculation, differentially expressed (DE) genes between lungs of infected and uninfected mice in each genotype with significant differences (FDR-adjusted P ≤ 0.05) and with more than 2.0-fold changes (n = 3 each) were determined. All DE genes are shown in Table E1. IPA was used to identify the canonical pathways that were enriched for these DE genes 60 days after MAC infection (Table E2). Table 1 shows the top 10 significantly enriched canonical pathways in infected wild-type lungs as compared with uninfected wild-type lungs. The majority of these 10 pathways were related to Th1 immunity, such as the ‘Th1 and Th2 Activation Pathway’ and the ‘Th1 Pathway’. Canonical pathways related to Th1 immunity were also significantly enriched in infected PD-1-deficient lungs compared with uninfected PD-1-deficient lungs, and they were enriched in infected PD-L1-deficient lungs compared with uninfected PD-L1-deficient lungs (Table 1). Principal-coordinate analysis (Figure E3A) showed two distinct large groups that were similar in all genotypes.

**Table 1:** The most significantly enriched canonical pathways compared between infected wild-type lungs and uninfected wild-type lungs 60 days after MAC infection.

Ingenuity canonical pathways	WT i vs WT c		PD-1 KO i vs PD-1 KO c		PD-L1 KO i vs PD-L1 KO c	
	P-value	% genes	P-value	% genes	P-value	% genes
Th1 and Th2 activation pathway	1.26E−22	46.1	1.26E−22	44.2	1.26E−19	42.2
Communication between innate and adaptive immune cells	3.98E−19	58.1	1.26E−21	59.5	3.98E−19	56.8
Th2 pathway	5.01E−19	47.1	1.58E−18	44.6	5.01E−15	41.3
Altered T cell and B cell signaling in rheumatoid arthritis	1.00E−17	53.7	3.16E−19	53.7	7.94E−18	52.4
Th1 pathway	3.16E−17	47.2	2.51E−17	45.4	3.16E−18	47.2
Dendritic cell maturation	3.16E−14	37.5	3.16E−14	35.6	3.98E−14	36.2
Neuroinflammation signaling pathway	1.00E−13	30.8	1.26E−17	31.9	3.98E−16	31.5
Primary immunodeficiency signaling	2.00E−13	63.4	2.04E−09	51.2	4.90E−06	41.5
T helper cell differentiation	3.98E−13	50.7	2.00E−13	49.3	7.94E−14	50.7
Agranulocyte adhesion and diapedesis	1.00E−12	35.3	2.51E−16	37.1	5.01E−15	36.5

*WT i* wild-type lungs infected with MAC during 60 days, *WT c* wild-type lungs treated with saline during 60 days, *PD-1 KO i* PD-1-deficient lungs infected with MAC during 60 days, *PD-1 KO c* PD-1-deficient lungs treated with saline during 60 days, *PD-L1 KO i* PD-L1-deficient lungs infected with MAC during 60 days, *PD-L1 KO c* PD-L1-deficient lungs treated with saline during 60 days.

**Figure 6:**
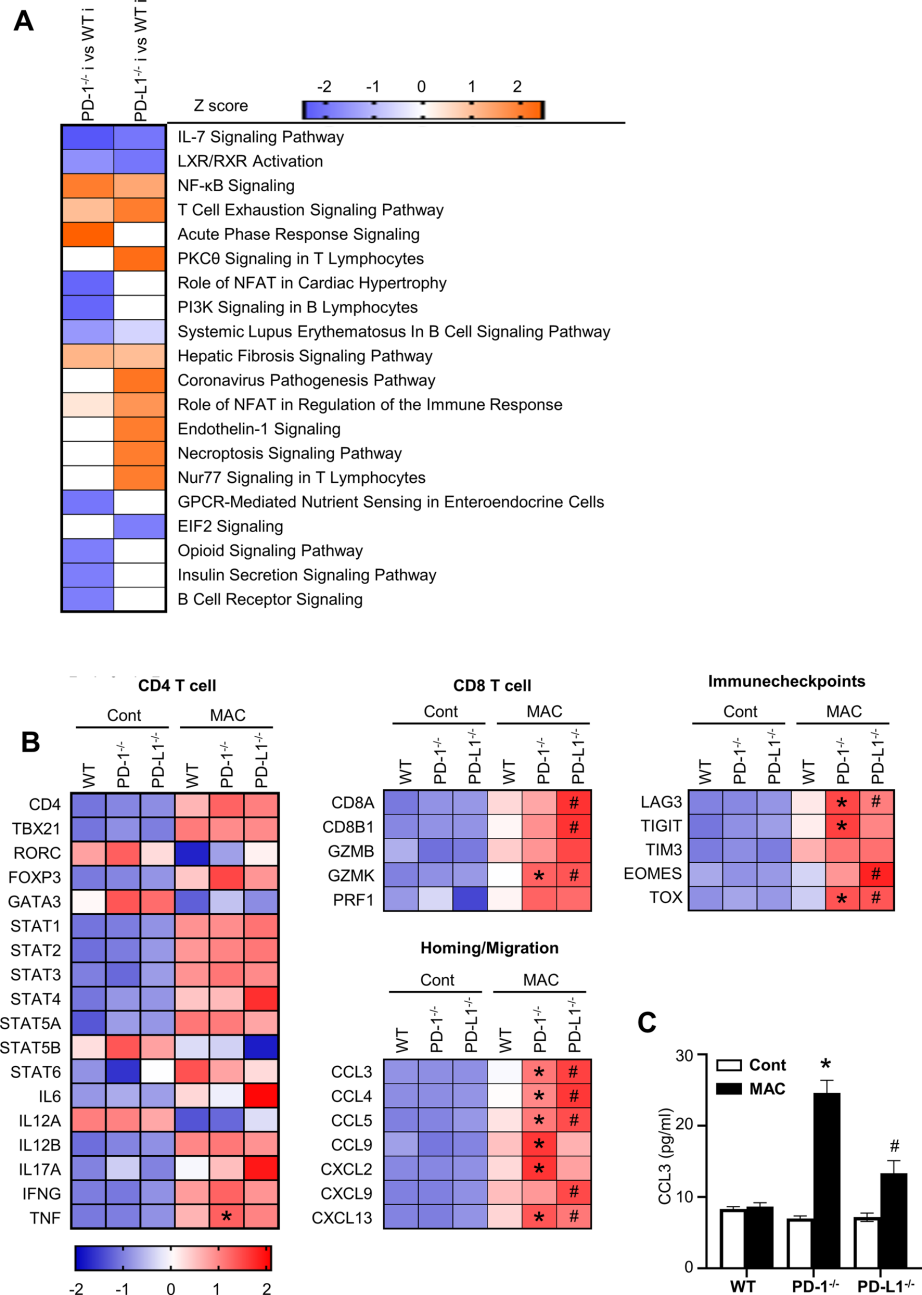
Differential expression of selected genes regarding CD4 T cells, CD8 T cells, chemokines, and immune checkpoints. (**A**) Top 20 significantly activated/repressed (Z score) pathways (adjusted-P value ≤ 0.05) identified by Ingenuity Pathway Analysis (Qiagen). Red, activated; blue, repressed. *WT i* wild-type lungs infected with MAC for 60 days, *PD-1*^*−/−*^ *i* PD-1-deficient lungs infected with MAC for 60 days, *PD-L1*^*−/−*^ *i* PD-L1-deficient lungs infected with MAC for 60 days. (**B**) Heatmaps of selected genes based on average expression values from each group. *Means significant difference between infected wild-type lung and infected *PD-1*^*−/−*^ lung (FDR-adjusted P ≤ 0.05 and with more than 1.2-fold changes, shown in Table E3). ^#^Means significant difference between infected wild-type lung and infected *PD-L1*^*−/−*^ lung (FDR-adjusted P ≤ 0.05 and with more than 1.2-fold changes, shown in Table E3). (**C**) CCL3 protein expressions in the BAL fluids of wild-type, *PD-1*^*−/−*^, and *PD-L1*^*−/−*^ mice 60 days after intranasal inoculation of 1 × 10^7^ CFU of MAC (solid bars). Control mice were administered saline (open bars). The experiments were performed in duplicate with four mice in each group. Data are expressed as means ± SEM. Significance was defined as a P value of < 0.05. *Significant difference between wild-type mice and *PD-1*^*−/−*^ mice (P < 0.05). ^#^Significant difference between wild-type mice and *PD-L1*^*−/−*^ mice (P < 0.05).

Therefore, considering that the effect of MAC infection itself on gene expressions was much greater than the effect of genotypes on them, gene expressions in mice infected with MAC were compared among genotypes to identify the effect of depletion of the PD-1/PD-L1 pathway. There were 281 DE genes with significant differences (FDR-adjusted P ≤ 0.05) and with more than 1.2-fold changes (n = 3 each; Table E3) between the infected lungs of *PD-1*^*−/−*^ and those of wild-type mice. There were also 352 DE genes between the infected lungs of *PD-L1*^*−/−*^ mice and those of wild-type mice (Table E3). The canonical pathways that were enriched for DE genes between the infected lungs of wild-type mice and those of *PD-1*^*−/−*^ mice and between those of wild-type mice and those of *PD-L1*^*−/−*^ mice are shown in Table E4. Figure 6A shows the top 20 significantly activated/repressed (Z score) pathways. Most pathways upregulated in the infected lungs of *PD-1*^*−/−*^ and *PD-L1*^*−/−*^ mice were related to inflammation involving T cell immunity such as “Acute Phase Response Signaling” and “T Cell Exhaustion Signaling Pathway” in *PD-1*^*−/−*^ mice and “PKCθ Signaling in T Lymphocytes” and “Nur77 signaling in T lymphocytes” in *PD-L1*^*−/−*^ mice.

To clarify the effects of PD-1/PD-L1 pathway depletion itself, gene expression in uninfected control mice was also compared between the lungs of *PD-1*^*−/−*^ mice and those of wild-type mice, and between *PD-L1*^*−/−*^ mice and those of wild-type mice. Overall, 95 and 214 genes were differentially expressed between uninfected lungs of *PD-1*^*−/−*^ mice and lungs of wild-type mice, and between uninfected lungs of *PD-L1*^*−/−*^ mice and lungs of wild-type mice, respectively [FDR-adjusted P ≤ 0.05, > 1.2-fold change (n = 3 each, Table E5)]. The DE genes between uninfected wild-type lungs and uninfected PD-1/PD-L1-deficient lungs were not related to the DE genes between infected wild-type lungs and infected PD-1/PD-L1-deficient lungs (Figure E3B). In addition, the DE genes in uninfected mice were not functionally relevant to the activation of CD8-positive cells, which suggested that the DE genes identified in our MAC infection model were not affected by the differences in the genotype itself.

The heatmaps using the selected DE genes related to CD4-positive T cells, CD8-positive T cells, and PD-1/PD-L1 pathways were created (Fig. 6B). Consistent with the findings from flow cytometry analyses, genes related to CD8-positive T cells, such as granzyme k (*GZMK*), were upregulated in the infected lungs of *PD-1*^*−/−*^ and *PD-L1*^*−/−*^ mice compared with those of wild-type mice. The expressions of *CD8A* and *CD8B1* were also upregulated in *PD-L1*^*−/−*^ mice. In addition, genes related to chemokines for T cell migration, such as *CCL3*, *CCL4*, *CCL5*, and *CXCL13*, were upregulated in the infected lungs of *PD-1*^*−/−*^ and *PD-L1*^*−/−*^ mice. The gene expression of *CCL3* was the most upregulated chemokine gene in the infected lungs of *PD-1*^*−/−*^ and *PD-L1*^*−/−*^ mice (Table E3, Fig. 6B), and the CCL3 protein level in the BAL fluids (Fig. 6C) was also higher in *PD-1*^*−/−*^ and *PD-L1*^*−/−*^ mice than in those of wild-type mice 60 days after MAC inoculation. In contrast, there were no differences in gene expressions related to CD4-positive T cells, such as *CD4*, *TBX21*, *RORC*, *FOXP3, GATA3* and *IFN-γ*, according to the genotype. As for immune checkpoint molecules related to T cell exhaustion other than PD-1 and PD-L1, lymphocyte-activation gene 3 (*LAG3*) and thymocyte selection-associated high-mobility group box (*TOX*) were upregulated in the infected lungs of *PD-1*^*−/−*^ and *PD-L1*^*−/−*^ mice. The gene expression of T-cell immunoreceptor with immunoglobulin and ITIM domain (*TIGIT*) was also upregulated in *PD-1*^*−/−*^ mice, whereas Eomesodermin (*Eomes*) was upregulated in *PD-L1*^*−/−*^ mice. To validate these transcriptome data, the expressions of *IFN-*γ, *GZMK*, *CCL3*, and *LAG3* genes were quantitatively analyzed by RT-PCR. It was confirmed that the changes in these gene expressions were similar to those obtained from RNA-seq analysis (Figure E4).

These findings suggest that depletion of the PD-1/PD-L1 pathway did not affect the activity of CD4 Th1 immunity, but rather activated CD8-positive T cell immunity in response to MAC infection. In addition, some molecules related to immune checkpoints seemed to be upregulated in a compensatory response in *PD-1*^*−/−*^ and *PD-L1*^*−/−*^ mice.

## Discussion

Although the PD-1/PD-L1 pathway has been shown to play a pivotal role in host defense against various infections including *M. tuberculosis*^8,9^, little is known about MAC infection^13^. This study was intended to examine the impact of depletion of the PD-1/PD-L1 pathway on pulmonary NTM disease using a mouse model, and it demonstrated that genetic depletion of PD-1 or PD-L1 did not affect mortality and the bacterial burden. The IFN-γ/IL-12 axis of Th1 immunity plays pivotal roles to kill MAC bacteria^14^. We previously reported that *T-bet*, which is a master regulator of Th1 cells, was critically associated with susceptibility to pulmonary MAC infection^15^. Consistent with our previous report, the present RNA-seq analysis showed that genes related to the Th1 pathway were most significantly enriched and upregulated in infected lungs of wild-type mice, and also in infected lungs of *PD-1*^*−/−*^ and *PD-L1*^*−/−*^ mice, with the expression levels of genes related to Th1 immunity similar among infected lungs of these three genotypes.

In the present model, depletion of the PD-1/PD-L1 pathway did not affect the activities of CD4 T cells. Since the expression of *LAG3* was upregulated in the infected lungs of *PD-1*^*−/−*^ and *PD-L1*^*−/−*^ mice, compensation by other inhibitory pathways, such as LAG3, may have suppressed exaggerated activation of CD4-positive T cells. In fact, PD-1/PD-L1 blockade was shown to upregulate LAG-3 or other immune checkpoints as a compensatory mechanism in tumor-bearing mouse models^16,17^. Furthermore, given that TOX initiates and dominates the development of exhausted T cells at the transcriptional and epigenetic levels^18^, the upregulated expression of *TOX* in the infected lungs of *PD-1*^*−/−*^ and *PD-L1*^*−/−*^ mice may have induced LAG-3 in the infected lungs of *PD-1*^*−/−*^ and *PD-L1*^*−/−*^ mice.

It has been thought that CD8-positive T cells regulate *M. tuberculosis* infection in both humans and mouse models^19,20^. In contrast, depletion of CD8-positive T cells was not found to lead to exacerbation of MAC disease^3,21^. In the present study, the expressions of genes related to CD8-positive T cells and multiple chemokines were significantly increased in *PD-1*^*−/−*^ and *PD-L1*^*−/−*^ infected lungs, although the increased number of cytotoxic CD8-positive T lymphocytes seemed to be not involved in bacterial control and mortality in the *PD-1*^*−/−*^ and *PD-L1*^*−/−*^ mice.

A previous study showed that CCL3 activates the migration of CD8-positive T cells, rather than CD4-positive T cells^22^. In the present study, CCL3 was the most prominent chemokine in the lungs of *PD-1*^*−/−*^ and *PD-L1*^*−/−*^ mice after MAC infection. Therefore, elevation of CCL3 in the infected lungs of *PD-1*^*−/−*^ and *PD-L1*^*−/−*^ mice may play some role in the recruitment of CD8-positive T cells in these mice. In addition, *Eomes* was upregulated in the infected lungs of *PD-L1*^*−/−*^ mice, which induces the differentiation of CD8-positive T cells into effector and memory phases^23^.

As for *M. tuberculosis* infection, it was demonstrated that blocking the PD-1/PD-L1 pathway exacerbates the infection. Worsening of *M. tuberculosis* infection was caused by administration of anti-PD-1 antibody to lung cancer patients^9^. In a mouse experiment, *PD-1*^*−/−*^ mice died soon after infection with *M. tuberculosis* showing high bacterial levels and excessive inflammation^8,24,25^. Excessive activation of Th1 immunity to *M. tuberculosis* is thought to be the mechanism that causes exacerbation of the infection^8,24,25^. On the other hand, the present study showed that blockade of PD-1/PD-L1 did not enhance the Th1 immune response against MAC, and there was no change in mortality or bacterial load after MAC infection. Differential involvement of the PD-1/PD-L1 pathway in Th1 immune responses between tuberculosis and nontuberculous mycobacterial infections was suggested.

This study has some limitations. The current study focused mainly on lung lymphocytes, but not on other immune cells, such as dendritic cells and macrophages^26^. The results of the present experiments were based on genetic depletion of PD-1 or PD-L1, which is not equivalent to antibody-mediated blockade in clinical settings in terms of the timing of blocking the PD-1/PD-L1 pathway^27^.

In conclusion, the present results demonstrated that depletion of PD-1 or PD-L1 did not affect Th1 immunity and the mortality of mice infected with MAC. However, blocking of the PD-1/PD-L1 pathway might cause lung inflammation by regulating the accumulation and activation of CD8-positive T cells during pulmonary MAC infection. This finding might imply that, when the PD-1/PD-L1 pathway is blocked in cancer patients who are also infected with MAC, more attention should be paid to the development of lymphocytic pneumonia than to the exacerbation of pulmonary MAC disease.

## Methods

All methods described in this study were performed in accordance with relevant guidelines and regulations.

### Mycobacteria

*M. avium* subsp. *hominissuis* was the same clinical isolate used in the previous report^15^ and was grown to mid-log phase in Middlebrook 7H9 liquid medium (Difco/Becton Dickinson), aliquoted, and frozen at − 80 °C until use. Bacterial counts in each organ were determined by plating serial dilutions of organ homogenates of individual mice onto Middlebrook 7H10 agar plates and counting bacterial colonies 2 weeks after plating.

### Mice and infection

All animal experiments were approved by the University of Tsukuba Institutional Animal Care and Use Committee. Wild-type C57BL/6 mice were purchased from Charles River. *PD-1*^*−/−*^ mice were provided by Dr. Tasuku Honjo^28^. *PD-L1*^*−/−*^ mice were generated at the Laboratory Animal Resource Center, University of Tsukuba^29^. Female mice (8–12-week-old) were infected with *M. avium* subspecies *hominissuis* via intranasal inoculation at a dose of 1 × 10^7^ colony forming units (CFU) in 50 μl of saline. Control mice were treated with 50 μl of saline. The study was carried out in compliance with the ARRIVE guidelines.

### Histology

Lung sections were stained with hematoxylin and eosin stain. Ziehl–Neelsen stain was used to detect bacilli.

### Immunohistochemistry

Paraffin-embedded sections of lung tissues were de-paraffinized with xylene and rehydrated through an ethanol series and PBS. A microwave pretreatment was applied for antigen retrieval. Endogenous peroxidase was blocked with 0.3% H_2_O_2_ in methanol for 30 min, followed by incubation with G-Block (Genostaff) and the avidin/biotin blocking kit (Vector). The sections were incubated with anti-CD4, CD8, granzyme B, and perforin (Cell Signaling Technology) antibodies at 4 °C overnight. For the subsequent reaction, biotin-conjugated anti-rabbit Ig (DAKO) and peroxidase-conjugated streptavidin (Nichirei) were used. Peroxidase activity was visualized by diaminobenzidine. The sections were counterstained with Mayer’s hematoxylin.

### Bronchoalveolar lavage (BAL)

Lungs were lavaged with six sequential 1-ml aliquots of saline. The first lavage was used to determine protein levels. Lactate dehydrogenase (LDH) levels in the first lavage were also assessed using an LDH cytotoxicity assay kit (Promega). The CCL3 concentration in the first lavage was determined by ELISA (R&D Systems). Cells were counted using a hemocytometer, and differential cell counts were obtained by staining with Diff-Quick (Polysciences, Inc.) after cytospins.

### Flow cytometry

Lungs were digested with 75 U/ml collagenase (type 1; Sigma) at 37 °C for 90 min, and isolated cells were filtered through 20-μm nylon mesh. Cells were then stained with anti-CD4, CD8, CD62L, and TCR-β antibodies (BioLegend) and analyzed by flow cytometry. Single cells and live cells were gated using FSC-W/FSC-A and SSC-A/Zombie yellow, respectively. Lymphocytes were gated by SSC/FSC, and the T cells were gated by TCR-β. To determine the absolute number of a cell population, cell counting beads were added in each sample with normalized volume. T cell production of intracellular IFN-γ was determined by flow cytometry using APC-conjugated anti-mouse IFN-γ (BioLegend), as described previously^15^.

### RNA extraction from lung tissues and sample preparation for RNA-seq

Total RNA from lung tissues was extracted using TRIZOL with a homogenizer. In 18 individual samples (3 from the lungs of the wild-type mice treated with saline, 3 from the lungs of the *PD-1*^*−/−*^ mice treated with saline, 3 from the lungs of the *PD-L1*^*−/−*^ mice treated with saline, 3 from the lungs of the wild-type mice infected with *M. avium* for 60 days, 3 from the lungs of the *PD-1*^*−/−*^ mice infected with *M. avium* for 60 days, and 3 from the lungs of the *PD-L1*^*−/−*^ mice infected with *M. avium* for 60 days), RNA quality was examined by the RNA 6000 Pico kit (Agilent, Santa Clara, CA). An amount of 500 ng total RNA was used for RNA-seq library preparation with the NEB NEBNext rRNA Depletion Kit and the NEBNext Ultra Directional RNA Library Prep Kit (New England Biolabs, Ipswich, MA); sequencing was performed with NextSeq500 (Illumina, San Diego, CA) by Tsukuba i-Laboratory LLP (Tsukuba, Ibaraki, Japan). FASTQ files were analyzed using CLC Genomics Workbench (CLC-GW, Version 10.1.1, Qiagen). Reads were mapped to mouse reference genome (mm10) and quantified for annotated genes. The Empirical Analysis of DGE tool in CLC-GW was used to detect differential expression of genes (false discovery rate ≤ 0.05, and fold change ≥ 2.0 or ≥ 1.2). The data are available under GEO series accession number GSE169202.

### Reverse transcription-polymerase chain reaction (RT-PCR)

Total RNA was extracted from lungs. Real-time quantitative RT-PCR was performed using QuantStudio 5 (Applied Biosystems). The PCR primers used in this study are listed in Table E6 in the supplemental material. The target gene expression levels were calculated using the ΔΔCT method and normalized against glyceraldehyde 3-phosphate dehydrogenase mRNA.

### Statistical analysis

Data are expressed as means ± SEM. Data comparisons among the experimental groups were performed using one-way ANOVA followed by post hoc tests. Survival data were analyzed by the Kaplan–Meier method and the log-rank test. Values of P ≤ 0.05 were considered significant. GraphPad Prism Version 7 (GraphPad Software Inc.) was used for the analyses.

### Ethics approval and consent to participate

All animal studies were approved by the Institutional Review Board.

### Consent for publication

All authors consent.

## Supplementary Information

Supplementary Figure 1.

Supplementary Figure 2.

Supplementary Figure 3.

Supplementary Figure 4.

Supplementary Legends.

Supplementary Table 1.

Supplementary Table 2.

Supplementary Table 3.

Supplementary Table 4.

Supplementary Table 5.

Supplementary Table 6.

## Data Availability

All data generated or analyzed during this study are included in this published article. As for raw data of RNA-seq, they are available under GEO series accession number GSE169202.

## Abbreviations

PD-1: Programmed cell death-1
PD-L1: Programmed cell death-ligand 1
MAC: *Mycobacterium avium–intracellulare* Complex
NTM: Non-tuberculous mycobacterial
ICI: Immune checkpoint inhibitors
PD-1^−^^/^^−^: PD-1-deficient
PD-L1^−^^/^^−^: PD-L1-deficient
BCG: *Mycobacterium bovis* Bacillus Calmette–Guérin
BAL: Bronchoalveolar lavage
LDH: Lactate dehydrogenase
LAG3: Lymphocyte-activation gene 3
TOX: Thymocyte selection-associated high-mobility group box
TIGIT: T-cell immunoreceptor with immunoglobulin and ITIM domain
Eomes: Eomesodermin
