# Supplementary figures and images for: Depletion of PD-1 or PD-L1 did not affect the mortality of mice infected with Mycobacterium avium
Source: Sci Rep. 2021 Sep 9;11:18008. doi: 10.1038/s41598-021-97391-4 (PMC8429769; doi:10.1038/s41598-021-97391-4)

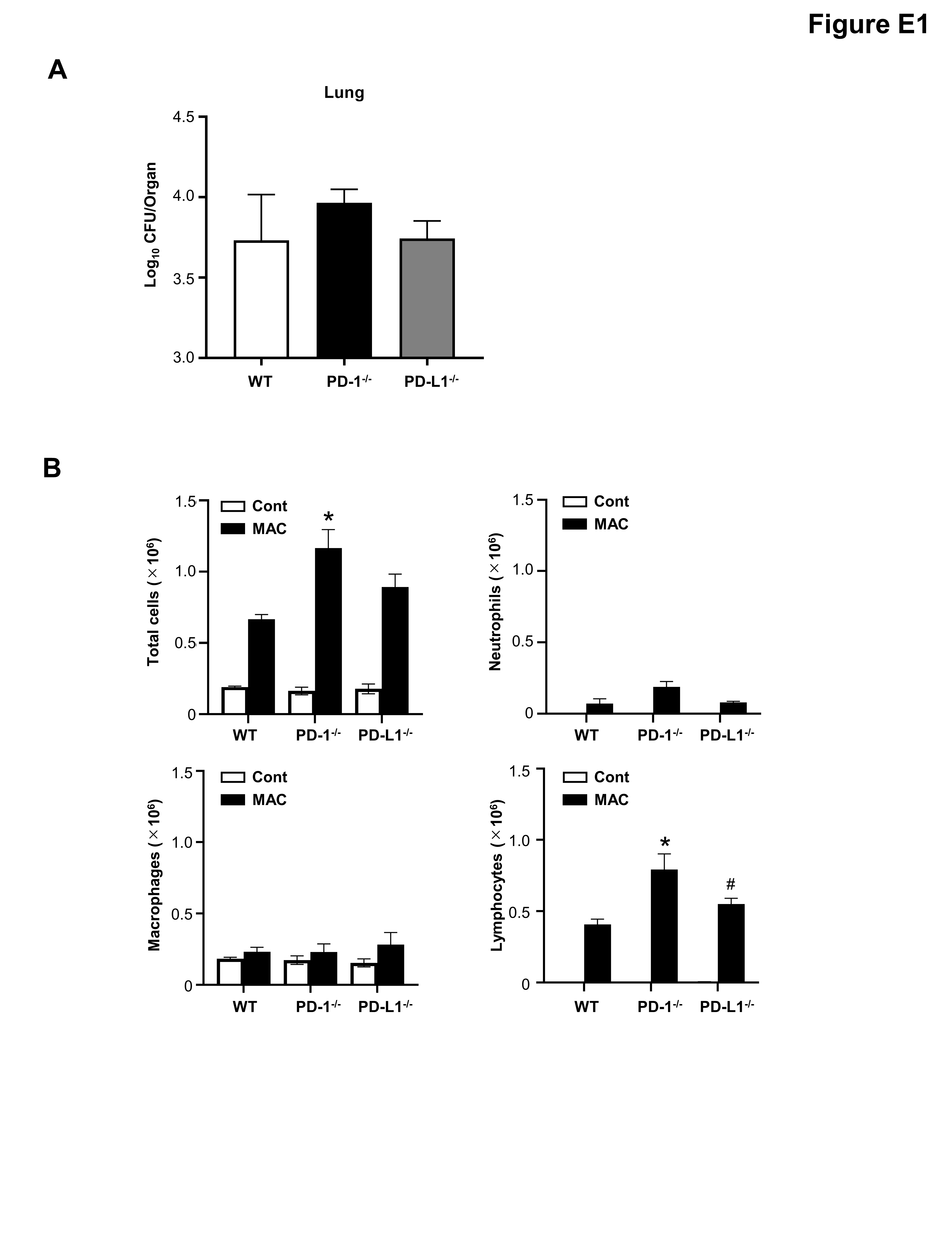

Supplement: Supplementary file 1 — Supplementary Figure 1. [file 41598_2021_97391_MOESM1_ESM.tif]

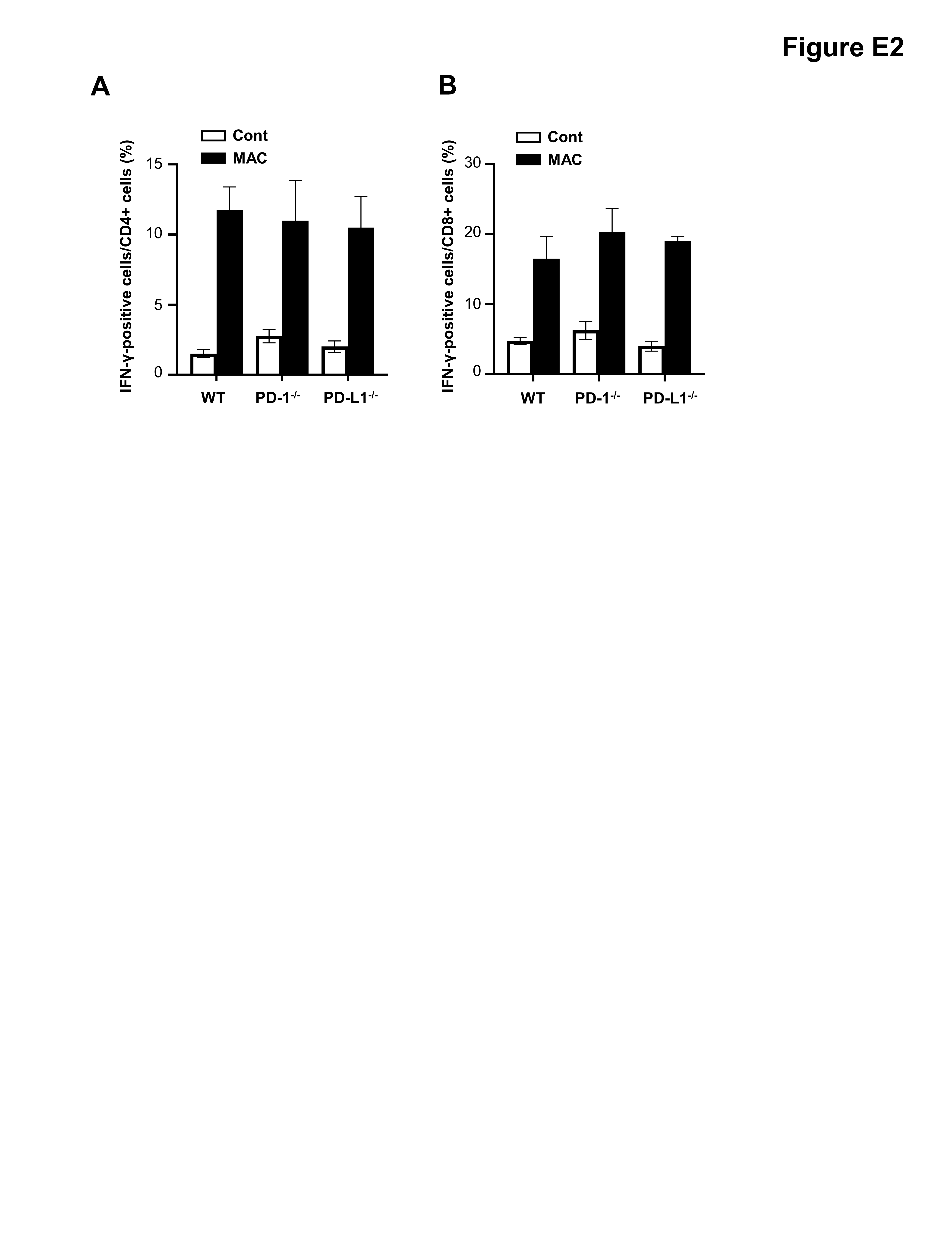

Supplement: Supplementary file 2 — Supplementary Figure 2. [file 41598_2021_97391_MOESM2_ESM.tif]

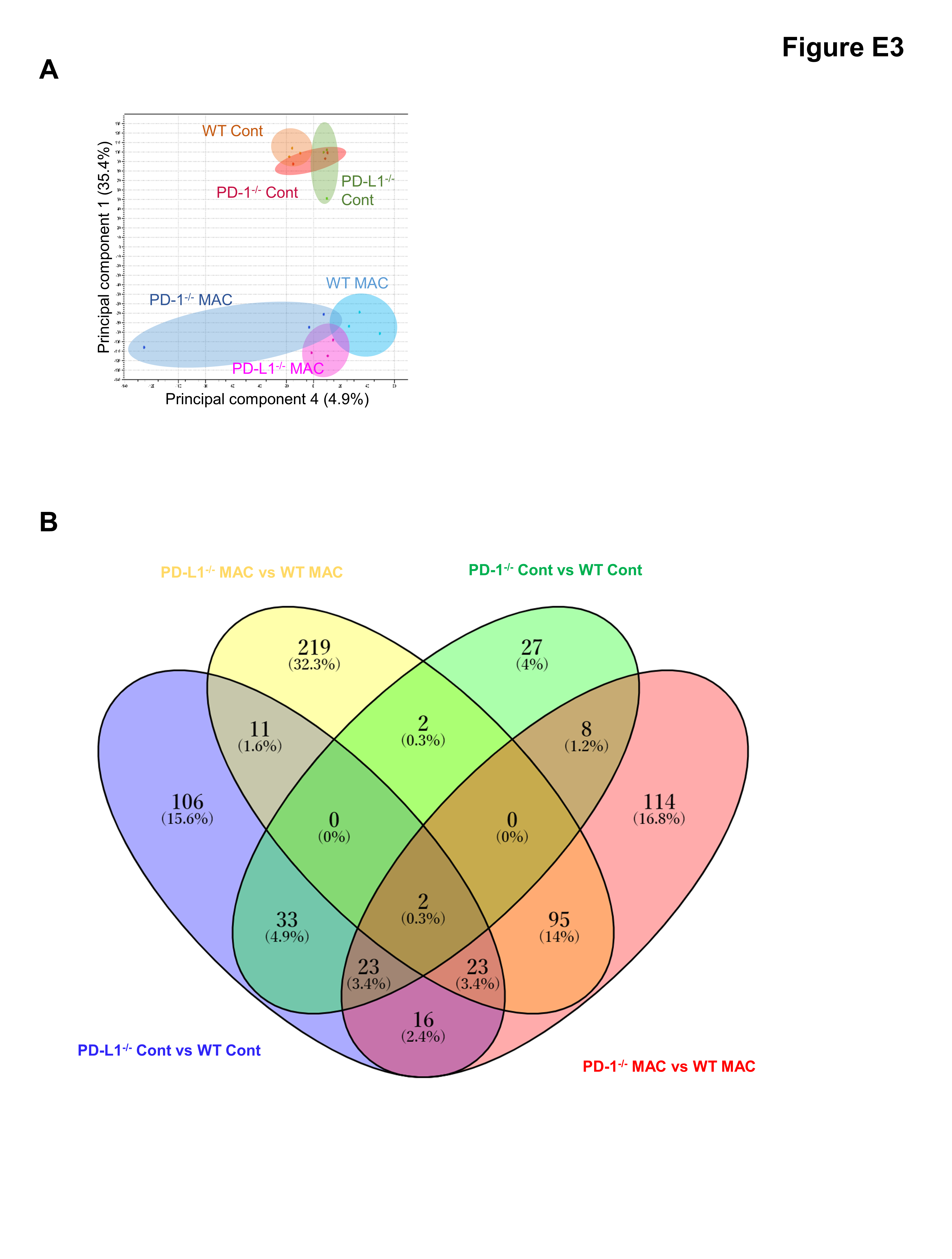

Supplement: Supplementary file 3 — Supplementary Figure 3. [file 41598_2021_97391_MOESM3_ESM.tif]

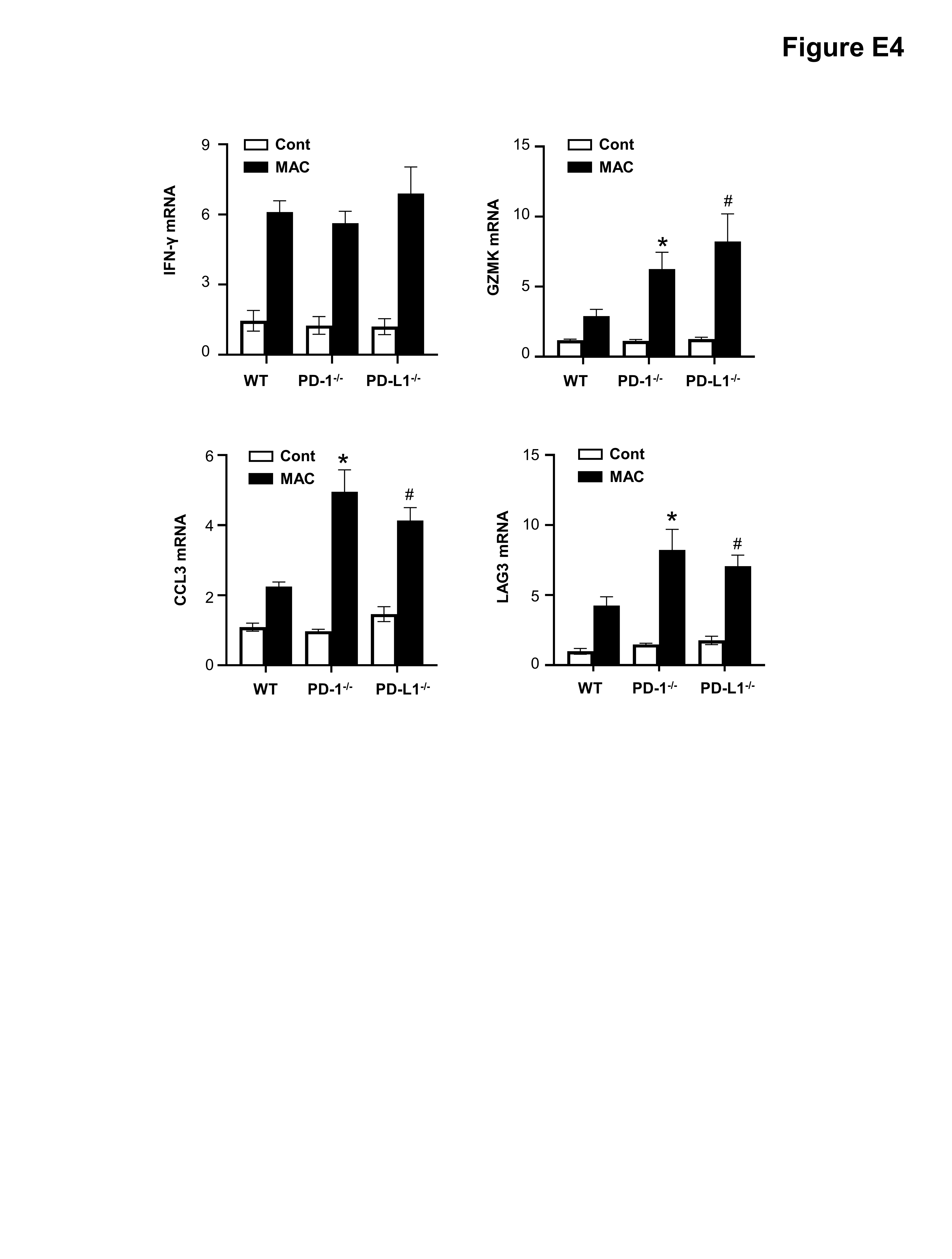

Supplement: Supplementary file 4 — Supplementary Figure 4. [file 41598_2021_97391_MOESM4_ESM.tif]
