# Supplementary material for: Depletion of PD-1 or PD-L1 did not affect the mortality of mice infected with Mycobacterium avium
Source: Sci Rep. 2021 Sep 9;11:18008. doi: 10.1038/s41598-021-97391-4 (PMC8429769; doi:10.1038/s41598-021-97391-4)
Supplement: Supplementary file 5 — Supplementary Legends. [file 41598_2021_97391_MOESM5_ESM.docx]

**Depletion of PD-1 or PD-L1 did not affect the mortality of mice infected with *Mycobacterium avium***

Masayuki Nakajima, Masashi Matsuyama, Mio Kawaguchi, Sosuke Matsumura, Takumi Kiwamoto, Yosuke Matsuno, Yuko Morishima, Kazufumi Yoshida, Mingma Thsering Sherpa, Kai Yazaki, Ryota Tanaka, Naoko Okiyama, Masafumi Muratani, Yukio Ishii and Nobuyuki Hizawa

Online Data Supplement

**Supplementary Figure legends**

**Supplementary Figure 1. Depletion of PD-1/PD-L1 did not affect the susceptibility to *M. avium* (1×10^5^ CFU), but affected pulmonary lymphocytic inflammation.**

**(A)** Mycobacterial outgrowth in the lungs of wild‑type, *PD-1^-/-^*, and *PD-L1^-/-^* mice 60 days after intranasal inoculation of 1×10^5^ CFU of MAC. The results are expressed as CFU per organ. The experiments were performed in duplicate with eight mice in each group. Data are expressed as means± SEM. Significance was defined as a P value of <0.05. **(B)** Numbers of total cells, neutrophils, macrophages, and lymphocytes in BAL fluids of wild-type, *PD-1^-/-^* and *PD-L1^-/-^* mice 60 days after intranasal inoculation of 1×10^5^ CFU of MAC. Control mice were administered saline (open bars). The experiments were performed in duplicate with four mice in each group. Data are expressed as means± SEM. Significance was defined as a P value of <0.05. *Significant difference between wild‑type mice and PD-1*^-/-^* mice (p<0.05). #Significant difference between wild-type mice and *PD-L1^-/-^* mice (p<0.05)**.**

**Supplementary Figure 2. IFN-γ expression in CD4 and CD8-positive T cells is not altered by the PD-1 pathway following MAC infection.**

The proportions of IFN-γ-producing cells in CD4-positive (A) or CD8-positive (B) T cells obtained from lungs of wild type, *PD-1^-/-^*, and *PD-L1^-/-^* mice 60 days after intranasal inoculation of 1×10^7^ CFU of MAC or saline (Cont). Experiments were performed in duplicate with four mice in each group. Data are expressed as means± SEM.

**Supplementary Figure 3. Results of RNA-seq show that the effect of MAC infection itself on gene expressions is far much greater than the effect of genotypes on them.**

(A) Principal-component analysis of gene expression patterns from individual mice with or without MAC infection for 60 days. (B) Venn diagram is shown. Overlap comparison of differentially expressed genes detected in *PD-1^-/-^* lungs versus wild-type lungs and *PD-L1^-/-^* lungs versus wild-type lungs between uninfected control and 60 days after MAC infection (FDR-adjusted P≤0.05 and with more than 1.2-fold changes).

**Supplementary Figure 4. Validation of RNA-seq data by RT-PCR.**

Expressions of *IFN-γ*, *GZMK*, *CCL3*, and *LAG3* in the lung of wild type, *PD-1^-/-^*, nd *PD-L1^-/-^* mice 60 days after intranasal inoculation of 1×10^7^ CFU of MAC (solid bars). Control mice were administered saline (open bars). Experiments were performed in duplicate with four mice in each group. Data are expressed as means± SEM. Significance was defined as a P value of <0.05. *Significant difference between wild type mice and *PD-1^-/-^* mice (p<0.05). #Significant difference between wild type mice and *PD-L1^-/-^* mice (p<0.05).

**Footnotes of Supplementary Tables**

**Supplementary Table 1.**

See attached Excel file. All differentially expressed (DE) genes in the MAC-infected lungs compared with MAC-uninfected lungs in each mouse genotype.

WT i: Wild-type lungs infected with MAC during 60 days, WT c: Wild-type lungs treated with saline during 60 days, PD-1 KO i: PD-1-deficient lungs infected with MAC during 60 days, PD-1 KO c: PD-1-deficient lungs treated with saline during 60 days, PD-L1 KO i: PD-L1-deficient lungs infected with MAC during 60 days, PD-L1 KO c: PD-L1-deficient lungs treated with saline during 60 days

**Supplementary Table 2.**

See attached Excel file. All significantly enriched IPA canonical pathways (p value threshold ≤0.05 using Fisher’s exact test) in the MAC-infected lungs compared with MAC-uninfected lungs in each mouse genotype.

WT i: Wild-type lungs infected with MAC during 60 days, WT c: Wild-type lungs treated with saline during 60 days, PD-1 KO i: PD-1-deficient lungs infected with MAC during 60 days, PD-1 KO c: PD-1-deficient lungs treated with saline during 60 days, PD-L1 KO i: PD-L1-deficient lungs infected with MAC during 60 days, PD-L1 KO c: PD-L1-deficient lungs treated with saline during 60 days

**Supplementary Table 3.**

See attached Excel file. All differentially expressed (DE) genes in the MAC-infected lungs of *PD-1^-/-^* mice or *PD-L1^-/-^* mice compared with those of wild-type mice.

WT i: Wild-type lungs infected with MAC during 60 days, PD-1 KO i: PD-1-deficient　lungs infected with MAC during 60 days, PD-L1 KO i: PD-L1-deficient lungs infected with MAC during 60 days

**Supplementary Table 4.**

See attached Excel file. All significantly enriched IPA canonical pathways (p value threshold ≤0.05 using Fisher’s exact test) in the MAC-infected lungs of *PD-1^-/-^* mice or *PD-L1^-/-^* mice compared with those of wild-type mice.

WT i: Wild-type lungs infected with MAC during 60 days, PD-1 KO i: PD-1-deficient　lungs infected with MAC during 60 days, PD-L1 KO i: PD-L1-deficient lungs infected with MAC during 60 days

**Supplementary Table 5.**

See attached Excel file. All differentially expressed (DE) genes in the uninfected lungs of *PD-1^-/-^* mice or *PD-L1^-/-^* mice compared with those of wild-type mice.

WT c: Wild-type lungs infected with saline during 60 days, PD-1 KO c: PD-1-deficient　lungs infected with saline during 60 days, PD-L1 KO c: PD-L1-deficient lungs infected with saline during 60 days

**Supplementary Table 6.**

Primers used for RT-PCR.
