# Supplementary material for: Depletion of PD-1 or PD-L1 did not affect the mortality of mice infected with Mycobacterium avium
Source: Sci Rep. 2021 Sep 9;11:18008. doi: 10.1038/s41598-021-97391-4 (PMC8429769; doi:10.1038/s41598-021-97391-4)
Supplement: Supplementary file 11 — Supplementary Table 6. [file 41598_2021_97391_MOESM11_ESM.docx]

**Table E6.** **Primers used for RT-PCR**

| **Primer Target** | **Sequence** |
| --- | --- |
| GAPDH | 5′-CCGCATCTTCTTGTGCAGTG-3′ (forward) |
|  | 5′-CGTTGATGGCAACAATCTCC-3′ (reverse) |
| IFN-γ | 5′-CACGGCACAGTCATTGAAAG-3′ (forward) |
|  | 5′-TCTGGCTCTGCAGGATTTTC-3′ (reverse) |
| GZMK | 5′-TGGCTGGCGTTTATATGTCTTC-3′ (forward) |
|  | 5′-GCTGCGGTACTGGATGGAC-3′ (reverse) |
| CCL3 | 5′-TTCTCTGTACCATGACACTCTGC-3′ (forward) |
|  | 5′-CGTGGAATCTTCCGGCTGTAG-3′ (reverse) |
| LAG3 | 5′-CTGGGACTGCTTTGGGAAG-3′ (forward) |
|  | 5′-GGTTGATGTTGCCAGATAACCC-3′ (reverse) |
